# Evolution from electrophysiologic to hemodynamic monitoring: the story of left atrial and pulmonary artery pressure monitors

**DOI:** 10.3389/fphys.2015.00271

**Published:** 2015-10-07

**Authors:** Deirdre M. Mooney, Erik Fung, Rahul N. Doshi, David M. Shavelle

**Affiliations:** ^1^Cardiovascular Institute, Maine Medical CenterPortland, ME, USA; ^2^Department of Medicine, Tufts University School of MedicineBoston, MA, USA; ^3^Keck Medical Center of USC, University of Southern CaliforniaLos Angeles, CA, USA; ^4^Department of Medicine, Dartmouth CollegeHanover, NH, USA; ^5^School of Public Health, Imperial College LondonLondon, UK

**Keywords:** heart failure, implantable hemodynamic monitor, thoracic impedance, left atrial pressure monitor, pulmonary artery pressure monitor, LAPTOP trial, CHAMPION trial

## Abstract

Heart failure (HF) is a costly, challenging and highly prevalent medical condition. Hospitalization for acute decompensation is associated with high morbidity and mortality. Despite application of evidence-based medical therapies and technologies, HF remains a formidable challenge for virtually all healthcare systems. Repeat hospitalizations for acute decompensated HF (ADHF) can have major financial impact on institutions and resources. Early and accurate identification of impending ADHF is of paramount importance yet there is limited high quality evidence or infrastructure to guide management in the outpatient setting. Historically, ADHF was identified by physical exam findings or invasive hemodynamic monitoring during a hospital admission; however, advances in medical microelectronics and the advent of device-based diagnostics have enabled long-term ambulatory monitoring of HF patients in the outpatient setting. These monitors have evolved from piggybacking on cardiac implantable electrophysiologic devices to standalone implantable hemodynamic monitors that transduce left atrial or pulmonary artery pressures as surrogate measures of left ventricular filling pressure. As technology evolves, devices will likely continue to miniaturize while their capabilities grow. An important, persistent challenge that remains is developing systems to translate the large volumes of real-time data, particularly data trends, into actionable information that leads to appropriate, safe and timely interventions without overwhelming outpatient cardiology and general medical practices. Future directions for implantable hemodynamic monitors beyond their utility in heart failure may include management of other major chronic diseases such as pulmonary hypertension, end stage renal disease and portal hypertension.

## Introduction

Cardiovascular disease remains the leading cause of death in the United States and worldwide (Lim et al., 2012; Santulli, 2013). Heart failure (HF) is a costly, challenging and highly prevalent medical condition with major public health concerns given the associated significant morbidity and mortality (Ramani et al., 2010). Hospitalization for acute decompensated heart failure (ADHF) is a sentinel event that signifies disease progression and an inability of the heart to maintain adequate hemodynamics for perfusion and function of vital organs. Nearly half of these patients are readmitted within 6 months (Jong et al., 2002) and/or deceased by 1 year (Adams et al., 2005). The lifetime risk of developing HF for Americans age 40 years or older is approximately 20% (Yancy et al., 2013) The incidence of new HF cases exceeds 650,000 annually (Yancy et al., 2013). Over one million hospitalizations are attributed to HF annually with an estimated cost of ~$20 billion for the United States health care system (Yancy et al., 2013; Sharma et al., 2015).

To contain costs and standardize management of patients hospitalized for ADHF, the Centers for Medicare and Medicaid Services have recently introduced regulations to withhold or reduce payments for unnecessary hospitalizations for HF (http://CMS.gov)^1^. Increasingly scrutinized are readmission rates and other performance metrics (e.g., length of hospital stay, medical regimen at discharge) that are fuelling efforts to reduce HF readmissions. Evidence based therapies, including optimal medical therapy with neurohormonal antagonists and implantable devices (e.g., cardiac resynchronization therapy, defibrillators), are well outlined by major cardiovascular and electrophysiological societies including the American College of Cardiology, American Heart Association, Heart Failure Society of America, European Society of Cardiology, European Heart Rhythm Association, and Heart Rhythm Society (Heart Failure Society of America et al., 2010; McMurray et al., 2012; Brignole et al., 2013; Russo et al., 2013; Yancy et al., 2013; Kusumoto et al., 2014). Despite application of these treatments and technologies, HF remains a formidable challenge for virtually all healthcare systems. Moreover, the quality of evidence for care, support and monitoring systems as well as the infrastructure to support HF patients, particularly in the outpatient setting, are lacking (Yancy et al., 2013).

The recent development and clinical trials of implantable hemodynamic monitoring devices hold promise to reduce HF hospitalizations, with the potential to improve patient outcomes. The limited but emerging supportive evidence is encouraging further efforts to improve the patient experience with this clinically challenging medical condition. Leading experts in this field have acknowledged the difficulty of conducting clinical trials using cardiac monitoring embedded with therapeutic management to effect “hard” clinical outcomes and endpoints (Abraham et al., 2014). They have also underscored the importance of careful clinical trial design, endpoint selection, outcome assessment, management of actionable results, and other ethical issues (Abraham et al., 2014). This review focuses on implantable hemodynamic monitors that evolved from electrophysiologic (EP) devices with extended functionality to dedicated standalone pulmonary artery pressure monitors (e.g., CardioMEMS™)^2^ for guiding medical management of hemodynamic and volume status in outpatients, with demonstrable effects on reducing hospital readmission.

## Historical background

Early and accurate identification of impending and active ADHF is of paramount importance. Daily weight monitoring is a low-cost, easily accessible method of monitoring HF patients both in and out of the hospital. Unfortunately, weight as a reference value is easily confounded by changes in diet and muscle mass that are not related to intravascular volume status or filling pressures (Wolfel, 2007) and previous studies have found that the estimated positive predictive value for these findings are generally poor (Lewin et al., 2005; Zhang et al., 2009; Abraham et al., 2011a,b). Furthermore, while telemonitoring and collaborative multidisciplinary outpatient care teams appear to be practical effective measures to improve the management of HF, randomized studies have yet to confirm this (Chaudhry et al., 2010; Bekelman et al., 2015). The clinical symptoms of dyspnea, orthopnea, weight gain, and leg edema are often late indicators of congestion and volume overload that may already warrant hospitalization. Physical examination maneuvers such as inspection of the jugular venous pressure waveform, hepatojugular reflux and the square wave sign are useful surrogate measures of cardiac filling pressures, however, inter- and intra-observer variability, inconsistent manifestations, and the need for the patient to present for a physical examination, limit their applicability to identify early decompensated heart failure in the outpatient setting (Drazner et al., 1999, 2008). In addition, the sensitivity and specificity of these signs and symptoms vary widely, depending on the clinical study (Stevenson and Perloff, 1989; McCullough et al., 2002).

A gold standard measure of congestion in HF is not overall volume status (Verbrugge et al., 2014), but rather the pulmonary artery occlusion pressure, also known as the pulmonary capillary wedge pressure (PCWP). The PCWP reflects left sided cardiac filling pressures and is measured by a pressure transducer on the end of a pulmonary artery catheter. An inflatable balloon on the distal portion of the catheter allows placement of the catheter into a sub-selected pulmonary artery (PA) branch. An elevated PCWP, exceeding 18–22 mmHg, indicates pulmonary edema and congestion. Given the potential dangers of an indwelling PA catheter for invasive hemodynamic monitoring, the patient is by convention required to stay in the intensive care unit.

**Figure 1:**
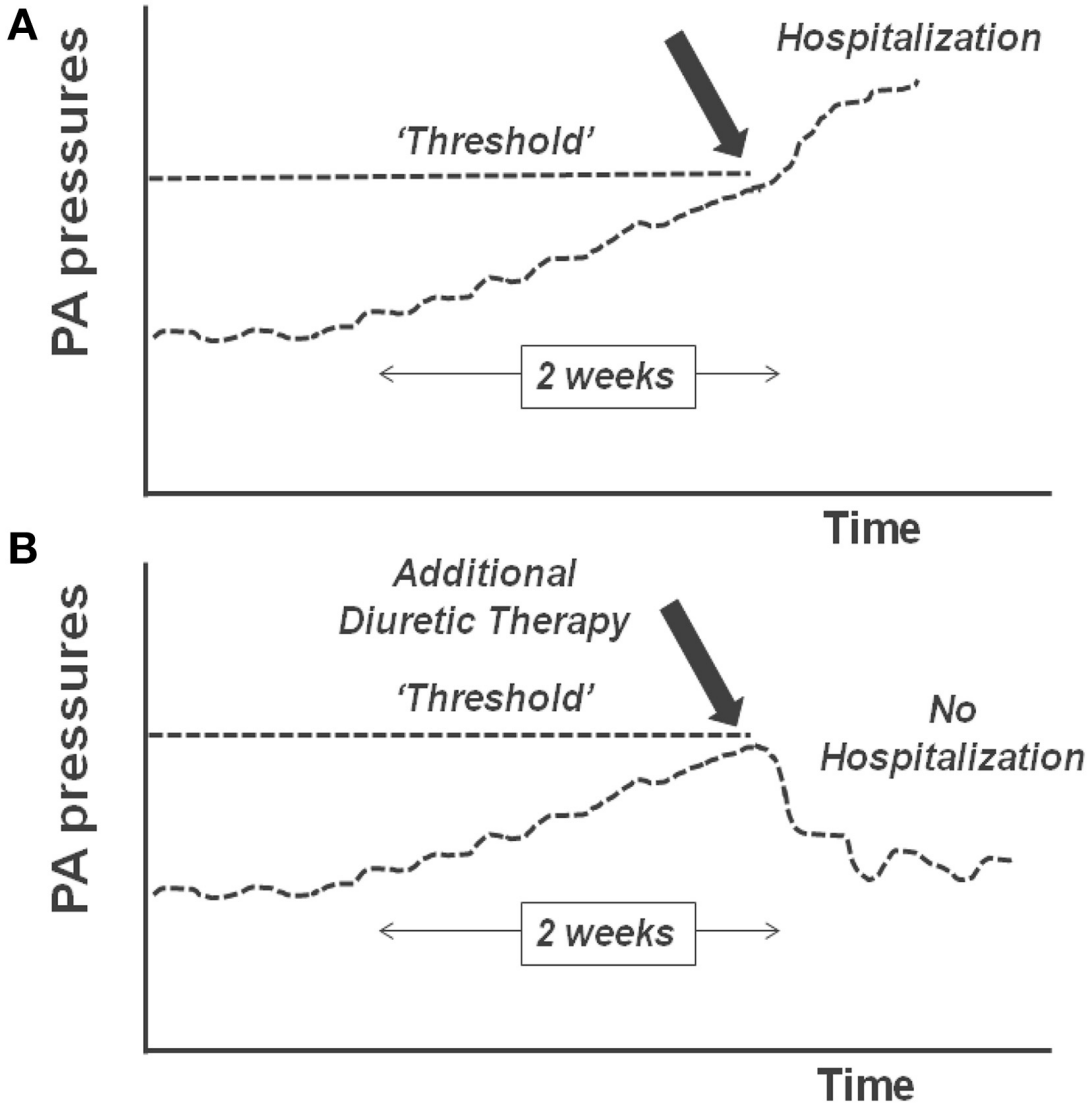
**(A)** Pulmonary artery (PA) pressures rise over time and cross a “threshold”; this results in decompensated heart failure and hospitalization. **(B)** When the rise in PA pressure is identified and additional diuretic therapy is given, the threshold is not crossed and hospitalization is avoided.

Advancement in medical microelectronics and the advent of device-based diagnostics have been developed to enable monitoring of ambulatory HF patients (Table 1). These devices transmit and report objective, quantitative data via remote monitoring systems. The premise of monitoring physiologic parameters is to enable clinicians to use these surrogate markers to optimize the patients' medical therapy in the ambulatory setting, before the onset of acute hemodynamic decompensation. This concept of remote device monitoring is also referred to as telemonitoring (Sousa et al., 2014). Several of the currently available telemonitoring systems measure various cardiac pressures and tailoring of medical therapy based upon these pressures is therefore called “pressure guided therapy.” The basis of pressure guided therapy involves the observation that most patients with HF require hospitalization because of excessive fluid accumulation. Accumulation of fluid occurs over several weeks and eventually reaches a “threshold” that requires hospitalization (Zile et al., 2008). Knowledge of these pressure increases can thus allow adjustment of medications to avoid reaching this “threshold” (Figure 1).

**Table 1:** **Device specifications and indications for use**.

**Device**	**Primary Indication**	**FDA Status**	**Primary measured variable(s)**	**Implant Location**	**Pertinent contraindications or restrictions**
OptiVol® Fluid Status Monitoring system (Medtronic, Inc., USA)	Ambulatory HF surveillance in patients who also meet indication for ICD therapy	Approved Nov 2004	Intrathoracic impedance and heart rate variability	Pectoral muscle region	Patients without an indication for ICD therapy or limited thoracic venous access
Chronicle® ICD and Chronicle® implantable hemodynamic monitor (Medtronic, Inc., USA)	Ambulatory HF surveillance in patients who also meet indication for ICD therapy	Not approved	RV systolic pressure, RV diastolic pressure (an estimate of PADP), maximum change in pressure over time (dP/dt and –dP/dt)	Right ventricle	Patients without an indication for ICD therapy or limited thoracic venous access
HeartPod® (St Jude Medical, Inc., USA)	Ambulatory HF surveillance	Not approved	Mean left atrial pressure	Left atrium	Patients unable to perform Valsalva maneuvers and maintain an airway pressure >39 mmHg for 8 s (required for periodic device calibration)
CardioMEMS™ HF System (CardioMEMS, Inc./St Jude Medical, Inc., USA)	Ambulatory surveillance in HF patients with NYHA III symptoms who have preserved EF or reduced EF on OMT, who have had a HF hospitalization in the previous year	Approved May 28, 2014	Systolic, diastolic, and mean pulmonary artery pressure	Left pulmonary artery (ideally, basal segmental branch)	Based on CHAMPION trial criteria, patient should not have any of the following: History of recurrent (>1) pulmonary embolism or deep vein thrombosis Inability to tolerate a right heart catheterization Recent major cardiovascular event (e.g., myocardial infarction, stroke) within 2 months of screening visit Recent CRT implanted ≤ 3 months prior to enrollment eGFR < 25 ml/min who are non-responsive to diuretic therapy or who are on chronic renal dialysis High likelihood of undergoing heart transplantation within 6 months of screening visit Congenital heart disease or mechanical right heart valve(s) Known coagulation disorders Hypersensitivity or allergy to aspirin, and/or clopidogrel

*CRT, cardiac resynchronization therapy; dP/dT, rate of rise of left ventricular pressure (mmHg/sec); EF, left ventricular ejection fraction; eGFR, estimated glomerular filtration rate; HF, heart failure; ICD, implantable cardioverter defibrillator; NYHA, New York Heart Association functional class; OMT, optimal medical therapy; PADP, pulmonary artery diastolic pressure; RV, right ventricle*.

**Figure 2:**
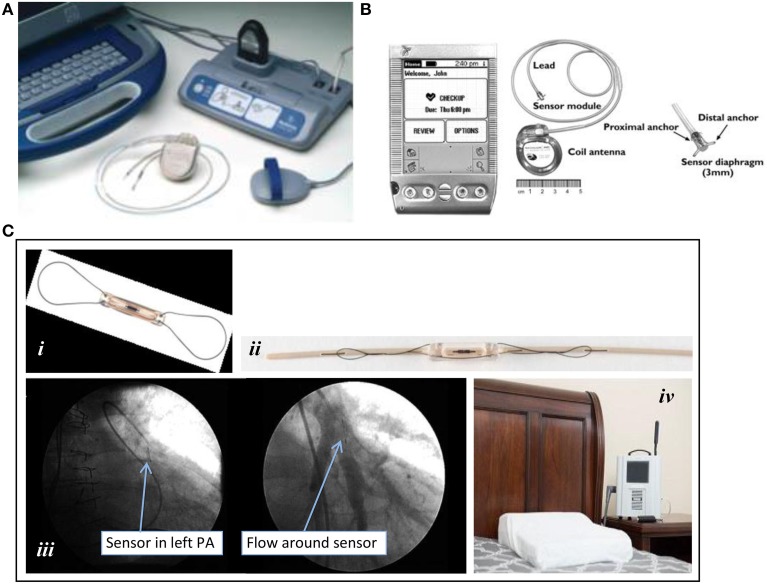
**Implantable ambulatory heart failure hemodynamic monitors. (A)** Chronicle®: (top left to bottom right) Implantable RV lead and ICD; lateral and posterior-anterior chest radiographs after device placement. **(B)** HeartPOD®. Implantable left atrial device and distal anchor. **(C)** CardioMEMS™ HF System: (i) pulmonary artery (PA) sensor; (ii) delivery catheter with preloaded PA sensor; (iii) pulmonary arteriograms showing a radiopaque PA sensor in a segmental branch of the left pulmonary artery (PA) before (left) and after (right) contrast dye injection; (iv) patient electronics system for transmission of data. All images were adapted with permission from Medtronic, Inc., St. Jude Medical, Inc., and Elsevier Inc.

Early investigational, implantable heart function monitoring devices piggybacked on the existing implantable cardioverter defibrillator (ICD) technology which had already established the safety of right ventricular pacing leads and was being used in the target population. Early devices used innovative transvenous lead technology to provide mixed venous oxygen saturation and pressures in the right ventricle (RV) (Ohlsson et al., 1996, 1998). The correlation between RV end diastolic pressure and PA end diastolic pressure was demonstrated. In addition, these devices also could measure additional physiologic parameters such as heart rate variability, body temperature, and other surrogates of patient activity levels (Raina et al., 2015). Information management varied and early models initially only provided real-time data during interrogations in the office. Later designs gained the capacity to store data and to transfer it securely and remotely. These devices culminated in the development of the Chronicle® IHM (IHM-2; Model 9520) (Figure 2A). The IHM-1 and IHM-2 devices have demonstrated significant changes in RV pressures associated with changes in diuretic therapy (Braunschweig et al., 2002), β-adrenergic receptor blockers (Ishikawa et al., 2009), biventricular pacing (Bruns et al., 2005), and inhaled therapies for pulmonary hypertension patients (Fruhwald et al., 2003; Karamanoglu et al., 2007).

Intrathoracic impedance monitoring was also evaluated as an adjunct to monitoring heart failure patients with an indication for an ICD or cardiac resynchronization therapy defibrillator (CRT-D) (Braunschweig et al., 2004; Yu et al., 2005). The OptiVol® function, an exclusive technology of Medtronic (Minneapolis, MN, USA) received the United States Food and Drug Administration (FDA) approval in 2004^3^. ICD devices with OptiVol can longitudinally monitor the conductance of a microelectrical current between the RV defibrillating coil and device case. When the fluid index sharply rises above the baseline in conjunction with a decrease in thoracic impedance, intrathoracic fluid accumulation such as pulmonary congestion is suggested. Elevated left ventricular (LV) filling pressure is associated with increased intrathoracic fluid (i.e., lung water), which in turn is associated with increased conductance and decreased impedance as this current travels across lung tissue. Other concomitant device data including heart rate variability, resting night heart rate, patient activity level, and the burden of atrial tachycardia or fibrillation noted around the time of changes in Optivol fluid index and thoracic impedance trends may help to improve confidence in interpretation of potentially actionable data. Initial trials demonstrated increased sensitivity for early detection of HF exacerbations with decreased unexplained alarms in comparison to the traditional weight based monitoring protocol (Abraham et al., 2011b).

Ambulatory monitoring of intrathoracic impedance has not had the clinical impact that was initially anticipated, with statistically non-significant results from several contemporary trials (Conraads et al., 2011; Yang et al., 2013) Contributing factors likely include the difficulty determining the difference between appropriate detection of pre-clinical events and false alarms, as well as the need for third party involvements to monitor trends and effect appropriate therapeutic changes. Threshold changes in impedance can also occur from non-cardiac etiologies (e.g., pneumonia, pneumothorax, positive pressure ventilation), pointing to the importance of interpreting those data in association with other concomitant device data, as well as clinical data from a (phone) discussion with the patient. Furthermore, monitoring of electrophysiology (EP) devices is traditionally far removed from those empowered to make changes in a patient's HF medications and arrange for appropriate follow up. A recent retrospective review of the data from the Fluid Accumulation Status Trial (FAST) and Program to Access and Review Trending Information and Evaluate Correlation to Symptoms in Patients With Heart Failure (PARTNERS-HF) trials by Abraham and colleagues suggested a novel scheme to stratify patients at risk for a HF related hospitalization using diagnostic physiologic monitoring parameters germane to most modern ICD devices (Sharma et al., 2015). Increasing numbers of device observations correlated with an increased risk of a HF hospitalization. However, as demonstrated in prior studies, rates of HF hospitalizations associated with alerts were low, around 14% for ≤ 3 observations.

In contrast to prior efforts that combined HF monitoring therapies with therapeutic EP devices, the left atrial pressure (LAP), and pulmonary artery pressure (PAP) ambulatory heart failure monitoring implantable devices were developed as purely diagnostic devices (Figures 2B,C). LAP monitoring was being explored around the same time that intrathoracic impedance was in the early post-marketing surveillance period (Ritzema et al., 2007). The LAP monitoring device incorporates direct left atrial pressure monitoring via a pressure transducer secured to the interatrial septum. Transvenous access and a transseptal puncture are required for implantation (see Table 2). Similar to early EP devices, LAP monitors (HeartPOD®, St. Jude Medical Inc, Sylmar, CA, USA) can transfer data through radiofrequency wireless transmissions done by direct interrogation of the coil antenna using the handheld patient advisory module (PAM®) (Figure 1B). The Hemodynamically Guided Home Self-Therapy in Severe Heart Failure Patients (HOMEOSTASIS) trial was published in 2011 and reported the safety, feasibility, accuracy, and reliability of LAP monitoring (Troughton et al., 2011). The Left Atrial Pressure Monitoring to Optimize Heart Failure Therapy Study (LAPTOP-HF) trial was designed to determine the safety and clinical effectiveness of a physician-directed, patient self-management therapeutic strategy, and has recently been completed; results are eagerly awaited (Maurer et al., 2015).

**Table 2:** **Procedural characteristics**.

	**LAP monitor**	**PAP monitor**
Device	HeartPOD®	CardioMEMS™
Access	Venous (femoral and subclavian vein)	Venous (usually femoral)
Approach	Transseptal puncture	Via PA
Accessories	Brockenbrough needle through 8 Fr sheath, 11 Fr delivery sheath in LA	12 Fr introducer sheath, dilators with access guidewire, 110-cm PA catheter, 0.018^′′^ x 260–300 cm fixed core guidewire with straight or angled tip
Intraprocedural anticoagulation	Heparin 5000 IU, intravenous	None
Imaging	Fluoroscopy, echocardiography (including TEE, TTE, ICE)	Fluoroscopy, pulmonary arteriography
Method and location of sensor deployment	Cinching and fixation of device anchors to inter-atrial septum	Release of preloaded sensor from over-the-wire delivery catheter
Associated implantable components	Coil antenna and lead	None
Duration of procedure	>1 h	20 min
Device interrogation	Transcutaneous detection of implanted sensor lead-antenna coil signal using handheld patient advisory module (PAM)	Transcutaneous detection of sensor-released energy in response to radiofrequency pulse from patient electronics unit
Post-procedural antithrombotics	Aspirin and warfarin for 30 days, then aspirin indefinitely	Aspirin and P2Y12 inhibitor (clopidogrel) for 1 month, then aspirin indefinitely; warfarin may substitute for aspirin after the first month if chronic anticoagulation therapy is required
Duration of implantation	Lifelong	Lifelong

*Fr, French gauge; IU, international units; LA, left atrium; PA, pulmonary artery; ICE, intracardiac echocardiography; TEE, transesophageal echocardiography; TTE, transthoracic echocardiography*.

Monitoring of PAP has been used for decades by cardiologists to detect early signs of HF in the intensive care setting (Rutherford et al., 1971). For ambulatory PAP monitoring, CardioMEMS (St. Jude Medical Inc, Sylmar, CA) was developed to directly measure systolic, diastolic and mean PAPs using a miniaturized wireless electromechanical sensor implanted in conjunction with a right heart catheterization procedure via transvenous access (Figure 1C). As the sole FDA-approved standalone device for outpatient HF monitoring, CardioMEMS was tested and proven to significantly reduce admissions for patients with New York Heart Association functional class III HF, regardless of left ventricular ejection fraction (LVEF) (Abraham et al., 2011a; Adamson et al., 2014), even in those with HF with preserved LVEF as opposed to those with reduced LVEF. A large post-approval trial is already recruiting (goal *N* = 1200) to verify the robustness, safety and usefulness of CardioMEMS in the complex real-world setting, particularly in reducing the rate of HF hospital readmissions and in improving patients' quality of life (clinicaltrials.gov).

## Deployment and monitoring

The Chronicle features a programmable device that bears resemblance to a pacemaker pulse generator, which is implanted to process and store information from the pressure sensor near the tip of the transvenous lead (Bourge et al., 2008). The device continuously records data such as heart rate, body temperature, estimated patient activity level, RV systolic and diastolic pressure, RV pressure changes, and estimated PA diastolic pressure. It is only programmed to store a smaller dataset based on programmed intervals, recording the median 6th and 94th percentile levels over that period. In the COMPASS-HF trial, patients were asked to use a handheld radio frequency device to transmit readings at least once weekly using a telephone line. Information was stored on a secure server that clinicians could access through a secure web site.

The device characteristics and key aspects of deployment of the HeartPOD and CardioMEMS are summarized in Table 2. The HeartPOD system consists of a microelectronic sensor and diaphragm housed in a cylindrical titanium casing (approximately 3 mm × 7 mm) equipped with deployment anchors, and linked with an implantable sensor lead and a coil antenna within a can that resembles a pacemaker (Figure 1B). After gaining femoral venous access, a Brockenbrough needle and transseptal sheath are advanced, and puncture of the interatrial septum is performed. Thereafter, a guidewire is introduced via a subclavian vein to secure the transseptal location and a delivery sheath is placed to allow for placement of the sensor lead and LA sensor. Once the correct position is confirmed with intracardiac or transesophageal echocardiography, the LA sensor with the implantable sensor lead is deployed. The sensor is oriented to the LA and is buttressed and immobilized permanently with proximal and distal nitinol anchors on the respective right and left atrial sides of the interatrial septum upon deployment. The electrode is then transferred from the femoral location to the infraclavicular position via an exchange catheter and attached to communication module. The metal alloy can, referred to as the implantable communications module or ICM, containing the coil antenna and microelectronics is implanted in the same manner as for a pacemaker. A prospective open-label observational study of 84 patients found that freedom from device failure was 95% at 2 years and 88% at 4 years (Troughton et al., 2011).

CardioMEMS is a battery-free, leadless sensor (15 mm × 3 mm) consisting of a coil and capacitor encased in silicone, with a nitinol wire loop at each end of the sensor (Figure 2Ci). The CardioMEMS device is preloaded on a delivery catheter with a tether release system (Figure 2Cii). The design of the system is based on microelectromechanical principles of resonance whereby an external antenna wand emitting radiofrequency energy can cause varying degrees of oscillations in the sensor depending on the ambient pressure. The implanting procedure requires a transfemoral venous approach for accommodation of a 12-French introducer sheath for the CardioMEMS delivery catheter (Table 2). PA catheterization is performed to document right sided pressures before and after device implantation. After identifying a posterior segmental branch of the left PA by selective pulmonary arteriography (Figure 2Ciii), the sensor is liberated as the tether release wire is pulled and withdrawn while the nitinol loops uncoil from the delivery catheter to maintain device position in the PA branch. Interrogation of PAP requires the patient to be in a supine position with the supplied pillow-like wand placed underneath the patient (Figure 2Civ). After approximately 20 s, systolic, diastolic and mean PAPs are measured and transmitted via wireless cellular network to the CardioMEMS data center. In the landmark CardioMEMS Heart Sensor Allows Monitoring of Pressure to Improve Outcomes in NYHA Class III Heart Failure Patients (CHAMPION) trial, the device- or system-related complication rate was only 1.4%, with an overall pressure-sensor failure rate of 0% (Abraham et al., 2011a).

Patients enrolled in the CHAMPION trial were asked to make daily measurements of their PAPs using their portable electronic unit and a special pillow containing an antenna to take daily sensor readings which are transmitted through a modem or cellphone to a secure patient database (Adamson et al., 2011). By requesting patients to lay on the special pillow, measurements should be more consistent reproducible and ideally leveled. In the LAPTOP-HF trial, LAP, body temperature, and intracardiac electrogram were measured. Subjects were able to power and interrogate their devices with radiofrequency wireless transmissions from their patient activator module or PAM (Ritzema et al., 2007), with capacity to store up to 3 months' data if 6 waveforms are acquired daily.

Both the HeartPOD and CardioMEMS systems use a physician-guided self-management model that is intuitive and conceptually sound. However, experts in the field have universally acknowledged the challenges in conducting implantable monitoring trials to demonstrate impacts on clinical outcomes, particularly with how the interrogated physiologic data are handled (Abraham et al., 2014). Monitoring of device data requires patient compliance, “physician compliance” and a structured action plan or algorithm in order to execute a successful program. Achieving the goal of reducing patient hospitalization and readmission for HF requires a team effort involving the patient, caretaker, primary care physician, cardiologist, nurse and/or support staff. As a team, they will need sufficient resources and training to appropriately interpret data trends. Standard easy to use protocols would lead to more uniform management and optimize the ability to study IHMs. These protocols will need to have some flexibility so health care providers can customize treatment plans to each individual as necessary. In monitoring data, it has been emphasized that data trends are more crucial to successful management than acting on individual abnormal data points. Potential harm could also be introduced with injudicious remote monitoring when treatments such as diuretic therapy and vasodilators are administered without careful consideration, understanding and interpretation of abnormal data. Establishing a good line of communication with the patient, and exercising clinical judgment (e.g., focused history taking to gather clinical cues, scheduling an outpatient visit when required, and assessing renal function and/or electrolytes after adjustment of medical therapy such as diuretics), may help to clarify abnormal and outlying data trends. An online secure website (https://cardiomemshf.com/user/sign_in) is accessible to healthcare professionals to view the interrogated data. The HeartPOD, CardioMEMS and other non-EP (i.e., without pacemaking or defibrillator functions) implantable monitoring systems have built-in, untapped features including cardiac output, heart rate variability, and electrocardiographic monitoring. If officially approved by the FDA, these additional monitoring parameters will likely improve characterization of hemodynamic derangements, and potentially reduce false-positive results, and associated resource utilization.

When using surrogate measures to direct therapy, it is crucial to understand exactly what is being measured. While mean PAP and LAP are both considered adequate surrogates for filling pressures, they are two different measurements and neither is the gold standard measurement (LVEDP). The PCWP is often considered to reflect left ventricular preload and pulmonary capillary hydrostatic pressure, however, there is ongoing debate about the validity of this assumption in the setting of various conditions including chronic pulmonary disease, mechanical ventilation and pulmonary venous scarring. In principal, the LAP would be a more accurate measurement as it is physically and physiologically closer to the gold standard, LVEDP, however it is more invasive to measure. A brief literature review did not reveal any studies comparing the three measurements simultaneously, however there are a few studies comparing LAP and PCWP. In 1962, the PAP, PCWP, and LAP measurements of 11 patients with either clinically normal hearts or suspected mitral valve disease were studied with right heart catheterization in a control state, during a norepinephrine infusion, and during positive and negative intraalveolar pressures (Luchsinger et al., 1962). This study demonstrated a strong linear relationship (*r* = 0.95) between PCWP and LAP in all settings with the PCWP being consistently 35% higher than the LAP. A more contemporary study of lightly sedated dogs reported that the mean PCWP accurately reflected LAP (Chaliki et al., 2002). In this study, mean PCWP again was highly correlated with LAP (*r* = 0.99; slope = 0.99; intercept = −0.46 mmHg). However, a study of 43 dogs and 30 patients in severe hemorrhagic, traumatic or septic shock noted that a dangerous rise in PAP was not reflected by PCWP or even central venous pressure (Hardaway, 1982). This discrepancy was attributed to suspected partial obstruction of the pulmonary microcirculation due to disseminated intravascular coagulation in the pulmonary venules. Central venous pressure should only rise due to high pulmonary pressures if there is RV failure.

With IHM, it is not only the sites from which data are collected but the manner in which they are recorded, stored and reported. In the HOMEOSTASIS trial, subjects were requested to make two LAP measurements a day with additional measurements during symptoms (Troughton et al., 2011). In the CHAMPION trial, continuous PAP measurements are recorded (Abraham et al., 2011a). Clearly, there are tradeoffs between the challenge of requiring patients in the real world to make multiple daily recordings using a separate handheld device and voluminous amounts of data that require no input from patients to collect.

## Clinical evidence for ambulatory monitoring implantable cardiovascular devices

As already seen with implantable cardiovascular devices, there is a wealth of data that can be harnessed through minimally invasive means and transmitted to a secure data repository via remote wireless technology. Newer implantable cardiac monitoring devices for HF offer the ability to provide individualized data trends and ideally predict clinical events before they occur. However, isolated device alerts need to be used in conjunction with other clinical data to avoid overutilization of health care resources and increased hospitalizations. Successful translation of remote device based monitoring into successful clinical management of these patients will require simple prospectively validated algorithms that indicate how to use raw data from individual devices to make timely and appropriate changes in clinical management without overburdening staff. At this time, despite a wealth of smaller studies evaluating these devices (Table 3), larger clinical radnomized trials are still necessary to demonstrate that implantable device based hemodynamic sensors beneficially impact morbidity and mortality in HF patients.

**Table 3:** **Available literature and clinical evidence on device efficacy**.

**Study (citation)**	**Study population**	**Objective**	**Formal outcomes**	**Study design**	**Key findings**	**Inclusion criteria**	**Exclusion criteria**
**OPTIVOL: INTRATHORACIC IMPEDANCE MONITORING**							
Fluid accumulation status trial (Abraham et al., 2011b)	*N* = 156, HFREF NYHA I–III symptoms with successfully implanted specific Medtronic ICD or CRT-D devices	Evaluate the sensitivity and unexplained detection rate associated with changes in intrathoracic impedance and with changes in daily weight and to compare the performance of these two measures	• Primary outcome: number of subjects with at least 30 days of daily impedance measurements; • Secondary outcomes included change in thoracic impedance associated with HF hospitalization for an exacerbation of HF or outpatient HF; number of adverse events	Multicenter non-randomized, prospective, double-blinded investigation	Increased sensitivity and decreased unexplained alarms in comparison to weight based protocol	• Subjects with one of the following ICDs: InSync Marquis™, InSync II Marquis™, Marquis® DR, or InSync III Marquis™ placed in the upper part of the left or right side of their chest • Subjects with a lead that is inserted through a vein and placed in the RV (a transvenous RV lead) • Subjects who underwent the ICD implant procedure, or any readjusting of the ICD, 30 days or more prior to enrolling in the study	• Enrolled in another clinical study • Received a heart transplant • Unable or unwilling to follow the study schedule of visits
OptiVol fluid index predicts acute decompensation of heart failure with a high rate of unexplained events (Yang et al., 2013)	*N* = 43; HFREF with NYHA III–IV on OMT undergoing Medtronic ICD or CRT-D implantation	Compare unplanned healthcare evaluation for a patient detected audible device alerts with or without proof of cardiac decompensation	Primary outcome: signs and symptoms of HF on physical examination and serologic evaluation	Prospective observational single site study	OptiVol fluid index had high sensitivity and high unexplained detection rate	Consecutive patients at a single center with HFREF (≤ 35%) NYHA III–IV on OMT for ≥3 months undergoing implantation of either a CRT-D (InSync Marquis 7298; Concerto C174AWK) or an ICD (Virtuoso VR D164VWC; Virtuoso DR D164AWG) from Sep. 2010 to Oct. 2012	• Life expectancy of less than 1 year • Anticipated difficulty in completing follow-up
Program to access and review trending information and evaluate correlation to symptoms in patients with heart failure (Partners-HF) (Whellan et al., 2010)	*N* = 694, patients with HF undergoing CRT-D implantation	Evaluate predictive ability of a monthly review of HF device diagnostic data to identify patients at higher risk for HF hospitalizations within 30 days	• Primary outcome: occurrence of HF related adverse event. • Secondary outcome: occurrence of HF related healthcare utilization, occurrence of HF related pulmonary congestion event	Prospective multi-center observational cohort study	Monthly review of HF device diagnostic data to identify patients at increased risk for HF hospitalizations within 30 days	• Meet ICD indications • NYHA III or IV • Receiving or have received a Medtronic CRT ICD within the previous 3 months • Able to sign and date informed consent • 18 years of age or greater • Available for follow-up visits, and be willing and able to comply with study protocol	• Acute MI, CABG or PTCA /stent within the last month • Mechanical right heart valve • Chronic (permanent) atrial arrhythmias • Life expectancy of less than 12 months • Status post-heart transplant • Undergoing kidney dialysis • Enrolled in a concurrent study that may confound the results of the study
Diagnostic outcome trial in heart failure DOT-HF (Van Veldhuisen et al., 2011)	*N* = 325, patients with NYHA II–IV HF	All-cause mortality or hospitalization for HF (time to first event)	• Primary endpoint: composite of all-cause mortality or heart failure hospitalization. • Secondary endpoints: all-cause mortality, the impact on total health care utilization, quality of life and cost effectiveness	Randomized open-label trial	Trial terminated early owing to slow enrolment and technological improvements; *post-hoc* futility analysis suggested positive result would have been unlikely	• HF NHYA II–IV • LVEF ≤ 35% • Indication for device implant according to ESC/AHA guidelines • A HF hospitalization or ED visit necessitating therapy within the past 12 months	• Post-heart transplant or actively listed on the transplant list and reasonable probability of undergoing transplantation in the next year • Received a CABG or valve surgery in last 90 days • MI in the last 40 days • Life expectancy < 1 year in the opinion of the physician • Severe COPD, as determined by physician and documented in medical records • Listed for valve replacement/valve repair • Severe, primary pulmonary hypertension • Serum creatinine ≥2.5 mg/dl measured within 14 days prior to enrolment • Chronic renal dialysis • Continuous or uninterrupted (≥2 stable infusions per week) infusion (inotropic) therapy for HF • Complex and uncorrected congenital heart disease
**CARDIOMEMS: PULMONARY ARTERY PRESSURE MONITOR**							
Comparison of a radiofrequency-based wireless pressure sensor to Swan-Ganz catheter and echocardiography for ambulatory assessment of pulmonary artery pressure in heart failure (Verdejo et al., 2007)	*N* = 12, NYHA II–IV	Correlation of PAP between wireless monitoring, PA catheterization and echocardiography at 0 and 60 days	Evaluate the accuracy of a new HF sensor, CardioMEMS, for PAP monitoring compared with PA catheterization and echocardiography in ambulatory HF patients at 0 and 60 days post-implantation	Single arm open enrolment with independent blind operators recording device measure-ments	Wireless PA monitoring correlated well with PA catheter and echocardio-graphic measurements	NYHA II–IV patients referred for ADHF with normal ventilation/perfusion lung scan and normal tricuspid regurgitation signal on echocardiography	• Recent ACS, CABG, or PTCA within last 3 months • Mechanical right heart valves Pulmonary or tricuspid stenosis • Documented pulmonary embolism • Pulmonary infarction within last 3 months • Pregnant • Active uncontrolled infection
CardioMEMS heart sensor allows monitoring of pressure to improve outcomes in NYHA class III heart failure patients (CHAMPION) trial (Abraham et al., 2011a)	*N* = 550, Patients with NYHA III HF with a HF admission within the past year, patients with low LVEF were on or started on OMT	6-month HF hospital admission rate	• Primary outcomes: rate of HF hospitalizations, and freedom from device failures • Secondary outcomes: change from baseline in mean PAP, proportion of patients hospitalized for HF, days alive outside of the hospital, quality of life	Prospective, multicenter, randomized, single-blind clinical trial	Patients allocated to the treatment arm had a significant reduction in HF related hospitalizations (84 vs. 120, HR 0.72, 95% confidence interval 0.60–0.65, *p* = 0.0002) with a NNT of 4 to prevent one HF hospitalization	• HF (HFpEF or HFrEF) ≥3 months • NYHA III • Subjects with HFrEF must be receiving a β-blocker for 3 months and an ACE-I or ARB for 1 month unless in the investigator's opinion, the subject is intolerant to β-blockers, ACE-I or ARB • At least 1 HF hospitalization ≤ 12 months of screening visit • PA branch diameter of 7–15 mm (implanted vessel)	• History of recurrent (>1) pulmonary embolism or deep vein thrombosis • Unable to tolerate a right heart catheterization • Major cardiovascular event (e.g., MI, CVA) ≤ 2 months of screening visit • CRT implanted ≤ 3 months prior to enrollment • eGFR < 25 ml/min who are non-responsive to diuretic therapy or who are on chronic renal dialysis • Likely to undergo heart transplantation ≤ 6 months of screening visit • Congenital heart disease or mechanical right heart valve(s) • Known coagulation disorders • Hypersensitivity or allergy to aspirin, and/or clopidogrel
Wireless pulmonary artery pressure monitoring guides management to reduce decompensation in HFpEF (Adamson et al., 2014)	*N* = 119, NYHA III patient with LVEF ≥40% enrolled in CHAMPION trial	6-month hospital readmission rate	6-month hospital readmission rate	Subgroup from a prospective, multicenter, randomized, single-blind clinical trial	50% reduction in hospitalization, more changes in diuretic and vasodilator therapies	See CHAMPION trial	See CHAMPION trial
**CHRONICLE: RIGHT VENTRICULAR PRESSURE AND OXYGEN SATURATION**							
The reducing decompensation events utilizing intracardiac pressures in patients with chronic heart failure (REDUCEhf) trial (Adamson et al., 2007, 2011)	*N* = 400. NYHA II–III patients with an indication for ICD and a previous HF hospitalization	Primary efficacy end point of HF hospitalizations, ED visits, or urgent clinic visits	Primary outcome: HF-related events (defined as hospitalizations >24 h or hospitalizations < 24 h requiring intravenous HF therapy, ED visits, or urgent clinic visits requiring IV therapy for HF) Primary safety end point: freedom from system-related complications at 6 months	Prospective, randomized, single blind (subject), parallel-controlled trial	Trial and enrollment stopped early due to lead failures in previous trials	• At least 18 years old • NYHA II or III • Clinically accepted indication for • ICD therapy • OMT • for at least 3 months prior to baseline • evaluation • At least one HF-related • event within the previous12 months	• Existing implantable CRM device (except a single-chamber ICD being considered for upgrade to a Chronicle ICD) • Indication for atrial pacing and/or CRT • Severe COPD, severe restrictive airway diseases; or primary pulmonary arteryhypertension • Known ASD or VSD Known tricuspid or pulmonary stenosis • Mechanical or bioprosthetic right heart valves • Severe, non-cardiac condition limiting 12-month survival • eGFR < 30 mL/min/1.73 m2 or on chronic renal dialysis • Likely to undergo cardiac transplantation within 12 months of implant • Receiving continuous or intermittent intravenous doses of vasoactive agents and/or positive inotropic therapy • Females of childbearing age not using reliable contraceptive measures
Chronicle offers management to patients with advanced signs and symptoms of heart failure (COMPASS-HF) (Bourge et al., 2008)	*N* = 274, NHYA III-IV patients with an indication for ICD; patients were on OMT for at least 3 months and had a HF hospitalization or ED visit within the preceding 3 months	Primary end points included failure, and reduction in the rate of HF-related events (hospitalizations and emergency or urgent care visits requiring intravenous therapy), freedom from system-related complications, freedom from pressure-sensor	• Primary outcome: efficacy of designated treatment strategies by demonstrating a reduction in the rate of all HF events in the treatment group compared to the control group • Primary safety end point: freedom from system-related complications and pressure sensor failure at 6 months • Secondary outcome: health care utilization, survival and days alive out of the hospital, rate of adverse events, predictive value of pressure change in the control group, quality of life, NYHA class, 6-min walk test performance	Prospective, multicenter, randomized, single-blind (subject), parallel-controlled trial	The Chronicle group had a non-significant 21% lower rate of all HF-related events compared with the control group (*p* = 0.33). A retrospective analysis of the time to first HF hospitalization showed a 36% reduction (*p* = 0.03) in the relative risk of a HF-related hospitalization in the Chronicle group	• NYHA III or IV • Managed with standard medical therapy for HF (such as diuretic, ACE-I or ARB, and β-blocker for at least 3 months prior to the baseline evaluation • At least one HF-related hospitalization or ED visit requiring intravenous treatment within 6 months prior to baseline evaluation	• Likely to be transplanted within 6 months from randomization or will remain hospitalized until transplantation • Severe COPD or restrictive airway disease • Continuous positive inotropic therapy • Known ASD or VSD • Mechanical right heart valves • Stenotic tricuspid or pulmonary valves • Presently implanted non-compatible pacemaker or ICD • CRT which has not achieved optimal programming for >3 months • Major cardiovascular event within 3 months prior to baseline evaluation • Severe non-cardiac condition limiting 6-month survival • Primary pulmonary artery hypertension • Serum creatinine greater than or equal to 3.5 mg/dL or on chronic renal dialysis • Enrolled in concurrent studies that may confound the results of this study • Pregnant or with child bearing potential and who are not on a reliable form of birth control
Direct left atrial pressure monitoring in ambulatory heart failure patients: initial experience with a new permanent implantable device (Ritzema et al., 2007)	*N* = 8; patients with NYHA III–IV symptoms and at least 1 HF hospitalization or unplanned visit for parenteral therapy in the last year	LAP correlation with simultaneous PCWP at 12 weeks	• Primary outcome: LAP correlation with simultaneous PCWP at 12 weeks • Primary safety end point: freedom from system complications	Multicenter, non-randomized, open-label feasibility clinical trial (first human experience with a permanently implantable, direct LAP monitoring system)	Ambulatory monitoring of direct LAP with a new implantable device was well tolerated, feasible, and accurate at a short-term follow-up	• Established HF • At least 1 HF hospitalization or unplanned visit for parenteral therapy in the last year • Ability to perform a modified Valsalva maneuver and achieve an airway pressure >40 mm Hg with an open glottis for >10 s	• Prior atrial septal surgery • PFO >2 mm; • Stroke or systemic thromboembolism within 6 months; • Chronic AF; • Atrial or ventricular thrombus; • Gastrointestinal bleeding in the last 6 months; • Requirement for chronic anticoagulation; or intolerance to aspirin, clopidogrel, or ticlopidine
Hemodynamically guided home self-therapy in severe heart failure patients (HOMEOSTASIS) trial (Troughton et al., 2011)	*N* = 84 patients with chronic severe HF; patients in phase 2 of enrollment had AF (*N* = 44)	LAP correlation with simultaneous PCWP at 3 and 12 months	Primary endpoints: LAP correlation with simultaneous PCWP at 3 and 12 months; freedom from Major Adverse Cardiac and Neurological Events at 6 weeks	Prospective, multicenter, observational open-label registry	LAP was highly correlated with simultaneous PCWP tracing; 82 out of 84 devices successfully implanted; 95% freedom from device failure	• Age >18 and < 85 • Documented history of HF with systolic or diastolic dysfunction of at least 6 months' duration • Patients with LVEF < 40% should receive maximally tolerated doses of ACE-I (or ARB if ACE-I is not tolerated), β-blockers, and anti-aldosterone therapy. The combination of hydralazine and nitrates should be considered in the persistently symptomatic African American patient • A history of NYHA II (OUS only), III or IV symptoms • Minimum of one (1) prior hospital admission within the last 12 months for exacerbation of HF or one (1) presentation to the ED or clinic requiring parenteral diuretic, vasodilator, inotrope, nesiritide, or equivalent treatment • Female subjects of childbearing potential must have a negative pregnancy test within seven (7) days before the procedure • Central venous vascular access • Capable of Valsalva maneuver with airway pressure >40 mm Hg for 10 s • The subject and the treating physician agree that the subject will comply with all required post-procedure follow-up, and that the patient is capable of correct device use as outlined in the protocol	• Intractable HF with resting symptoms despite maximal medical therapy or active listing for cardiac transplantation (< 6 months' survival expected) • Resting SBP < 90 or >180 mmHg • Acute MI, unstable ischemic syndrome within the last 6 weeks • PCI or cardiac surgery performed or planned within 6 weeks • Coexisting stenotic valve lesions, vegetations, hypertrophic cardiomyopathy, amyloidosis or other infiltrative heart disease, constrictive, restrictive disease, tamponade, or moderate or large pericardial effusion • History of deep venous thrombosis or pulmonary embolism • Surgical correction of congenital heart disease involving atrial septum • CVA or TIA within 6 months. History of uncorrected cerebral vascular disease • Atrial or ventricular thrombus, tumor or systemic thromboembolism • Symptomatic bradyarrhythmia or sustained VT/VF unless successfully treated with CRM device for 6 weeks • ASD or PFO >2 mm in diameter
							• Life expectancy < 1 year from malignancy, primary pulmonary hypertension, renal, hepatic, or neurological condition, etc • Gastrointestinal bleeding during the last 6 months • Coagulopathy or uninterruptible anticoagulation therapy or unable to take antiplatelet medications • Creatinine >2.5 gm/dl • Temperature >37.8^*oC*^ or WBC >13,000/mm^3^ • Currently participating in an investigational drug or another device study that has not completed the primary endpoint or that clinically interferes with the current study endpoints
Left atrial pressure monitoring to optimize heart failure therapy (LAPTOP-HF) (Maurer et al., 2015)	Plan for *N* = 730 patients with NYHA III symptoms and a HF hospitalization for elevated BNP within the last year	Safety and clinical effectiveness of a physician-directed, patient self-management therapeutic strategy based on LAP measured twice daily by a standalone implantable sensor or CRT-D compatible sensor, compared with a control group receiving OMT	• Primary outcome: reduction in relative risk of HF hospitalization • Primary safety end point: freedom from system complications at 12 months	Prospective, multicenter, randomized, controlled clinical trial	Ongoing, not recruiting participants	• Have ischemic or non-ischemic cardiomyopathy with either a history of reduced or preserved LVEF and HF for at least 6 months • NYHA III documented at screening visit • Be receiving appropriate medical therapy for HF as per ACC/AHA guidelines for at least 3 months prior to the randomization visit • Minimum of one (1) prior hospital admission within the last 12 months for acute exacerbation of HF of at least one (1) calendar date change duration requiring intravenous or invasive HF therapy. If CRT device previously implanted, the HF hospitalization must be ≥30 days after CRT implantation. Alternatively, if patients have not had a heart failure hospitalization within the prior 12 months, they must have an elevated BNP level of at least 400 pg/ml or an N-terminal pro-BNP (NT-proBNP) level of at least 1,500 pg/ml, according to local measurement at the time of screening (within 30 days of the screening visit/consent) Provide informed consent for study participation and be willing and able to comply with the required tests, treatment instructions and follow-up visits	• Age < 18 years • Pregnant • Intractable HF with resting symptoms despite maximal medical therapy • Patients listed for cardiac transplantation who are not likely to be transplanted within 12 months and who have improved to NYHA III without outpatient intravenous vasoactive medications or a VAD are eligible for the study, if they meet the other inclusion/exclusion criteria • Resting SBP < 80 or >180 mmHg • Acute MI, ACS, PCI, new CRM device (pacemaker, ICD, and CRT), CRM system revision, lead extraction or cardiac or other major surgery within 40 days • Coexisting, untreated, hemodynamically severe stenotic valve lesions, vegetations, hypertrophic cardiomyopathy with significant resting or provoked subaortic gradient, acute myocarditis, tamponade, or large pericardial effusion • ASD or PFO (with more than trace shunting on color Doppler or intravenous bubble study) or surgical correction of significant congenital heart disease involving atrial septum such as PFO or ASD closure device • CVA or TIA within 6 months • Inadequate vascular access for device implantation • 2D echocardiographic evidence of, or history of, unresolved left atrial or ventricular thrombus • Recent (within 6 months) or persistent deep venous thrombosis, pulmonary or systemic thromboembolism • Life expectancy < 1 year due to another illness • Coagulopathy or uninterruptible anticoagulation therapy or contraindication for all of the forms of antiplatelet/anticoagulant treatments anticipated in the protocol • eGFR < 30 ml/min/1.73 m^2^ by the MDRD method • Liver function test > 3 times upper limit of normal • Severe pulmonary disease producing frequent hospitalizations for respiratory distress and requiring continuous home oxygen • Pulmonary hypertension with a PASP ≥ 80 mm/Hg on screening echocardiogram • Active infection requiring systemic antibiotics • History of active drug addiction, active alcohol abuse, or psychiatric hospital admission for psychosis within the prior 2 years • Currently participating in a clinical investigation that includes an active treatment arm • Unable to demonstrate understanding and capability of using the patient advisory module, or PAM, appropriately • Does not have access to a telephone line usable for remote Patient Activator Module follow-up or electrical outlet for recharging the Module

*ACC/AHA, American College of Cardiology/American Heart Association; ACE-I, angiotensin converting enzyme inhibitor; ACS, acute coronary syndrome; ADHF, acute decompensated heart failure; AF, atrial fibrillation; ARB, angiotensin receptor blocker; ASD, atrial septal defect; BNP, B-type natriuretic peptide; CABG, coronary artery bypass grafting; COPD, chronic obstructive pulmonary disease; CRM, cardiac rhythm management; CRT-D, cardiac resynchronization therapy-defibrillator; CVA, cerebrovascular accident; ED, emergency department; eGFR, estimated glomerular filtration rate; ESC, European Society of Cardiology; HF, heart failure; HFpEF, heart failure with preserved left ventricular ejection fraction; HFrEF, heart failure with reduced left ventricular ejection fraction; HR, hazard ratio; ICD, implantable cardioverter defibrillator; LAP, left atrial pressure; LVEF, left ventricular ejection fraction; MI, myocardial infarction; NNT, number needed to treat; NYHA, New York Heart Association functional class; OMT, optimal medical therapy; PAP, pulmonary artery pressure; PASP, pulmonary artery systolic pressure; PCI, percutaneous coronary intervention; PCWP, pulmonary capillary wedge pressure; PFO, patent foramen ovale; PTCA, percutaneous transluminal angioplasty; RV, right ventricle; SBP, systolic blood pressure; TIA, transient ischemic attack; VAD, ventricular assist device; VT, ventricular tachycardia; VF, ventricular fibrillation; VSD, ventricular septal defect; WBC, white blood cell count*.

## Future of ambulatory heart failure implantable cardiovascular monitoring devices

Future directions for remote implantable PAP and LAP devices are broad. In cardiac patients, one can easily imagine the role for these devices in better understanding exercise physiology. They could also aid in clarifying the hemodynamics in particularly challenging outpatients such as those with difficult to assess pulmonary pressures by echocardiography (e.g., rheumatic mitral valve disease, severe pulmonary hypertension, morbidly obese patients.) Furthermore, in advanced HF patients with known arrhythmias, there can be a role to assess the clinical impact of supraventricular arrhythmias such as atrial fibrillation and ventricular arrhythmias, as well as addressing the question of whether these rhythm disturbances are causal or secondary to ADHF. Additionally, with the pressure to avoid indwelling lines, invasive procedures and overburdening intensive care units, pre-existing internal devices that monitor filling pressures could facilitate the management of these particularly high risk patients when admitted for both cardiac and non-cardiac issues, including perioperative hemodynamic and fluid management.

In advanced HF patients with left ventricular assist devices (LVAD) who are recurrently admitted with symptoms of congestion and fluid overload, LAP and PAP monitors may potentially help to discern elevated left sided filling pressures from other causes of dyspnea, and volume overload (e.g., chronic kidney disease progression, hypoalbuminemia, protein-losing enteropathy). However, further clinical review and evaluation still may be necessary to exclude a failing right ventricle in response to LVAD placement and manage other non-cardiac etiologies for recurrent hospitalizations. These devices may also be able to detect low filling pressures in patients with LVADs who urgently need increased intravascular volume in order for optimal device function and cardiac output. It remains to be seen whether regulatory agencies and transplantation societies will endorse the use of implantable LAP or PAP monitors as an alternative to indwelling PA catheters in the pre-heart transplant setting, with the intent to obviate the need for hospitalization in the intensive care unit and periodic replacement of PA catheters that are associated with procedural and other risks including line infection, sepsis, and thromboembolism.

There are also innumerable non-cardiac scenarios in which continuous assessment of cardiac hemodynamics and filling pressures would be invaluable. A recently published substudy of the CHAMPION trial found that of the 217 patients who did not meet criteria for pulmonary hypertension during the implantation right heart catheterization, 48.8% (*N* = 16) met criteria based on continuously observed PAP over the first week post-implantation. This implies that an implantable heart monitor may assist with improved diagnosis of pulmonary hypertension and perhaps better guide future trials targeting pulmonary hypertension (Frantz et al., 2008). It is foreseeable that future clinical investigations using these hemodynamic monitors may extend to non-HF patients, especially in efforts to improve management of volume status in the outpatient setting, improve patients' quality of life, and reduce rates of hospital readmission for hypo- or hypervolemia. Patients with end stage renal disease, primary pulmonary hypertension or portal hypertension are patient populations with similar high healthcare utilization. Renal replacement and diuretic therapies usually target a patient's known “dry weight,” which, as discussed above, is often not an accurate or reliable measure of true volume status. Furthermore, implantable hemodynamic monitors can detect other clinically significant events, such as poorly tolerated arrhythmias or hemodynamic shifts, that may be affecting patients and were previously unappreciated (Braunschweig et al., 2006).

## Author contributions

The review was initially conceived of and manuscript outlined by all authors. DM prepared the first draft in collaboration with EF. RD and DS provided substantial revisions and contributions. DS contributed Figure 1. The final version was reviewed and approved by all authors.

## Conflict of interest statement

Rahul N. Doshi has served as a consultant for St. Jude Medical, Inc. David M. Shavelle is a consultant and received research support from St Jude Medical, Inc. Deirdre M. Mooney and Erik Fung declare that the research was conducted in the absence of any commercial or financial relationships that could be construed as a potential conflict of interest.

## Footnotes

^1^CMS.gov. http://www.cms.gov/Medicare/Medicare-Fee-for-Service-Payment/AcuteInpatientPPS/Readmissions-Reduction-Program.html. Last accessed May 14, 2015.

^2^CardioMEMS HF System Post-Approval Study. NCT02279888. https://clinicaltrials.gov/ct2/show/study/NCT02279888?term=cardiomems&rank=1.

^3^U.S. Food and Drug Administration announcement, recently-approved devices. http://www.fda.gov/MedicalDevices/ProductsandMedicalProcedures/DeviceApprovalsandClearances/Recently-ApprovedDevices/ucm400550.htm. Last accessed: March 30, 2015.
